# Engineering Two-Phase Bifunctional Oxygen Electrocatalysts with Tunable and Synergetic Components for Flexible Zn–Air Batteries

**DOI:** 10.1007/s40820-021-00650-2

**Published:** 2021-05-15

**Authors:** Yanli Niu, Xue Teng, Shuaiqi Gong, Mingze Xu, Shi-Gang Sun, Zuofeng Chen

**Affiliations:** 1 Shanghai Key Laboratory of Chemical Assessment and Sustainability, School of Chemical Science and Engineering, Tongji University, Shanghai, 200092 People’s Republic of China; 2 State Key Lab of Physical Chemistry of Solid Surface, College of Chemistry and Chemical Engineering, Xiamen University, Xiamen, 361005 People’s Republic of China

**Keywords:** Bifunctional electrocatalysts, Oxygen electrocatalysis, Zn–air battery, Co/CoFe heterointerface engineering, Density functional theory calculations

## Abstract

Metal–air batteries, like Zn–air batteries (ZABs) are usually suffered from low energy conversion efficiency and poor cyclability caused by the sluggish OER and ORR at the air cathode. Herein, a novel bimetallic Co/CoFe nanomaterial supported on nanoflower-like N-doped graphitic carbon (NC) was prepared through a strategy of coordination construction–cation exchange-pyrolysis and used as a highly efficient bifunctional oxygen electrocatalyst. Experimental characterizations and density functional theory calculations reveal the formation of Co/CoFe heterostructure and synergistic effect between metal layer and NC support, leading to improved electric conductivity, accelerated reaction kinetics, and optimized adsorption energy for intermediates of ORR and OER. The Co/CoFe@NC exhibits high bifunctional activities with a remarkably small potential gap of 0.70 V between the half-wave potential (*E*_1/2_) of ORR and the potential at 10 mA cm^‒2^ (*E*_*j*=10_) of OER. The aqueous ZAB constructed using this air electrode exhibits a slight voltage loss of only 60 mV after 550-cycle test (360 h, 15 days). A sodium polyacrylate (PANa)-based hydrogel electrolyte was synthesized with strong water-retention capability and high ionic conductivity. The quasi-solid-state ZAB by integrating the Co/CoFe@NC air electrode and PANa hydrogel electrolyte demonstrates excellent mechanical stability and cyclability under different bending states.

**Supplementary Information:**

The online version contains supplementary material available at 10.1007/s40820-021-00650-2.

## Highlights

- A novel heterostructured bimetallic Co/CoFe nanomaterial supported on nanoflower-like N-doped graphitic carbon (NC) is prepared through a strategy of coordination construction-cation exchange-pyrolysis.
- The Co/CoFe@NC exhibits high bifunctional activities with a remarkably small potential gap of 0.70 V between oxygen evolution reaction (OER) and oxygen reduction reaction (ORR), which can be used in liquid and flexible quasi-solid-state rechargeable Zn–air batteries.
- The density functional theory calculations reveal optimized adsorption energies for intermediates of ORR and OER on heterostructured Co/CoFe@NC.

**Supplementary Information:**

The online version contains supplementary material available at 10.1007/s40820-021-00650-2.

## Graphical abstract

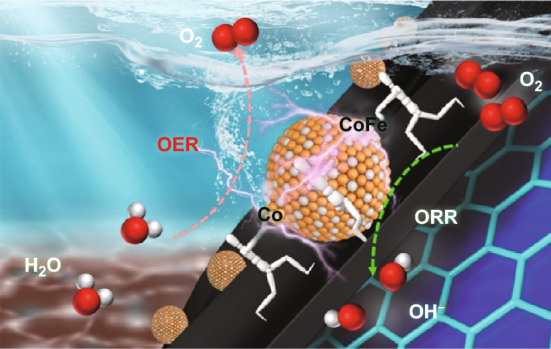

## Introduction

The rapid development of portable and wearable electronics has triggered intensive research activities on various energy conversion and storage devices [1, 2]. Metal–air batteries, especially flexible Zn–air batteries (ZABs) have been considered as promising candidates owing to their high theoretical specific energy density (1084 Wh kg^−1^), source abundance in nature, environmental benignity and high safety [3, 4]. Nevertheless, the wide application of the ZABs is still hampered by the sluggish kinetics of oxygen reduction reaction (ORR) and oxygen evolution reaction (OER) at the air cathode during the discharging and charging processes, arising from the multistep and proton-coupled electron transfer characters of the reversible oxygen electrocatalysis [5–7]. Currently, the noble metal Pt and metal oxides RuO_2_/IrO_2_ are the most accepted benchmark electrocatalysts for ORR and OER, respectively. However, these precious metal-based catalysts are plagued with high cost, severe scarcity and chemical susceptibility. In addition, their insufficient catalytic bifunctionality and inferior durability are also the shot-slab [8–10]. It is thus imperative to develop highly efficient bifunctional non-precious metal catalysts for rechargeable ZABs.

Under the alkaline condition, the discharge/charge (ORR/OER) reactions at the air cathode of ZABs are: O_2_ + 2H_2_O + 4e^−^ $\leftrightharpoons$4OH^−^. The ideal bifunctional catalysts can mediate this reversible oxygen reactions from equilibrium status to OH^−^ or O_2_ to a great extent [4, 11, 12]. Among various electrocatalysts ever reported, the material composites encompassing transition metal-based compounds (such as oxides, sulfides, carbides, and nitrides) supported on heteroatom-doped carbon (especially N-doped carbon) were explored as a class of compelling catalysts toward both ORR and OER [13–16]. The carbon support with unique porous structure is essentially important for the high performance of the composite catalysts, which can not only facilitate the charge transfer and mass transport, but also provide large surface area to ensure abundant exposed active sites. Recently, the supported bimetal alloy (e.g., CoFe, NiCo, and FeNi) composites have received growing interest owing to their binary active metal sites in a single nanoparticle, which may provide more selectivity for different catalytic reactions. In addition, the interaction between different metals can effectively modify the electronic structures, resulting in stabilized surface energy and moderate oxygen binding affinity [17–20].

At present, various strategies have been applied to synthesize bimetal alloy composite catalysts. High-temperature pyrolysis of the mixture of metal salts and nitrogen-rich small molecules provides a straightforward approach to prepare bimetal alloys supported in N-doped carbon (NC) [21–23]. Although the one-step pyrolysis of the mixture is a facile approach, the control of nanoparticle size and structure has become challenging. Moreover, the nanocrystals are prone to aggregate into bulk phase during the carbonization at high temperatures, leading to decreased electrocatalytic performance. Whereas the pyrolysis at low temperatures could moderate agglomeration, it would lead to low degree of graphitization and poor contact between active metal nanoparticles and underlying carbon, which also reduces the activity and stability of the catalyst materials [20, 24]. The thermal decomposition of metal–organic frameworks (MOFs) provides another appealing approach to prepare bimetal alloys supported in N-doped carbon. MOFs can be utilized as excellent self-sacrificial templates in view of their tunable central metals, abundant heteroatoms, and uniform porous structures. However, the integration of two or more metals into a single MOF is usually difficult because of the mononuclear metal center of most simple MOFs [25]. Alternatively, bimetal alloy composites may be obtained through a dual-MOF pyrolysis approach, which however increases the complexity of experiments and the inhomogeneity of electrocatalysts [26]. The rational design and construction of high-performance bifunctional oxygen electrocatalysts of bimetal alloys is still quite challenging for rechargeable ZABs.

To fabricate flexible ZABs, the solid-state electrolyte (i.e., hydrogel electrolyte) is an essential component, which largely governs the transport behavior of conductive ions and cycling stability of flexible ZABs. The low water take-up and retention, weak interaction with electrodes, and structural instability intrinsically associated with common polymer electrolytes, e.g., polyvinyl alcohol (PVA), polyethylene glycol, and gelatin have significantly limited their performance in solid-state batteries [27, 28]. Recently, a novel low-cost polyelectrolyte comprising sodium polyacrylate hydrogel (PANa) was developed for application in solid-state batteries. As a promising candidate of hydrogel electrolytes, the PANa-based electrolyte exhibits superior properties of water retention, ionic conductivity, electrode/electrolyte contact, and mechanical strength [4, 28]. However, the compatibility of PANa-based electrolyte with various air cathodes, especially under different bending states for achieving decent cyclability still presents a significant challenge for the practical application in flexible ZABs.

In this work, a novel Co-based coordination framework with uniform nanoflower structure was explored as precursor to prepare heterostructured Co/CoFe nanoparticles embedded in N-doped graphitic carbon. The preparation strategy is facile and versatile, which allows hydrothermal coordination reaction for morphology construction and subsequent cation exchange for composition regulation. The pyrolytic Co/CoFe@NC material delivers multiple advantages in terms of oxygen electrocatalysis for its unique morphology, heterostructure and bimetallic composition. It maintains the nanoflower morphology of the precursor, which can expose abundant catalytic active sites and enlarge catalyst/electrolyte contact area. The supportive and protective NC thin layer not only ensures a high electrical conductivity, but also prevents nanoparticles from agglomeration and dissolution during catalysis. The density functional theory (DFT) calculations suggest that the incorporation of Fe atoms can moderate the adsorption free energies of oxygen-containing intermediates in ORR and OER, which effectively boost the intrinsic activity of Co/CoFe@NC. The theoretical analysis also reveals the charge transfer from the metal layer to the NC layer, which can cause an electron-rich state on the latter and induce extra surface catalysis from carbon matrix. As a result, the optimized catalyst displays extraordinary bifunctional oxygen electrocatalysis with a small ∆*E* (*E*_*j*=10 _− *E*_1/2_) value of 0.70 V, which is among the best in literature reports. The rechargeable ZABs, including liquid batteries and flexible quasi-solid-sate batteries with a home-made sodium polyacrylate hydrogel (PANa) as electrolyte were assembled, demonstrating very impressive performance with high open-circuit voltage, small discharge/charge voltage gap and excellent long-term cyclability, and mechanistic flexibility.

## Results and Discussion

### Preparation and Physicochemical Characterizations

**Fig. 1:**
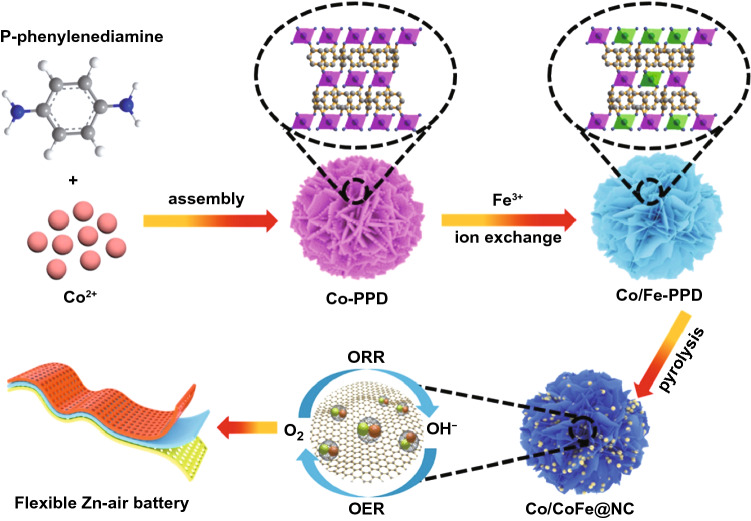
Schematic illustration of the synthetic route for Co/CoFe@NC

The synthesis process of Co/CoFe@NC can be divided into three major steps as illustrated in Fig. 1. Initially, a convenient solvothermal method was utilized to prepare Co-based coordination framework (denoted as Co-PPD) via the strong coordination interaction between the Co^2+^ ions and the amine functional group in *p*-phenylenediamine (PPD). As shown in Fourier transform infrared (FTIR) spectra (Fig. S1a), the peaks of Co-PPD at high wavenumber that arise from the stretching vibrations of the -N–H group become weaker as compared with the pure PPD. It signifies that the metal ions are anchored into the “nitrogen pots” in the Co-PPD [29]. In the X-ray diffraction (XRD) pattern of Co-PPD (Fig. S1b), only a small hump peak appears, suggesting the amorphous nature of the material. The scanning electron microscopy (SEM) images clearly show the nanoflower structure of Co-PPD comprised of numerous nanosheets (Figs. S1c, d). The corresponding X-ray spectroscopy (EDS) elemental mapping reveals the coexistence of C, N, and Co elements and their homogenous distribution in the whole sample (Figs. S1e-h).

In the following step, Co/Fe_*x*_-PPD was synthesized by a cation exchange of the as-prepared Co-PPD coordination framework. A series of Co/Fe_*x*_-PPD with different Fe contents were obtained by varying the amount of added Fe(NO_3_)_3_ in the solution bath for cation exchange. After thermal pyrolysis at 800 °C in Ar atmosphere, the Co/Fe_*x*_-PPD nanoflowers were transformed into heterostructured Co/CoFe_*x*_ nanoparticles embedded in N-doped porous carbon nanosheets (In the following study, the Co/CoFe@NC signifies a sample with optimized content of Fe, while the Co/CoFe_L_@NC and Co/CoFe_H_@NC denote samples with low and high contents of Fe, respectively). During this pyrolysis process, metal ions were reduced to the metallic state through a carbothermal reduction reaction with the organic ligands, which in turn can catalyze the growth of graphitic carbon layers on their surface. As contrast samples, the monometallic Co@NC, Fe@NC and undecorated NC catalysts were also prepared through a similar method (see the Supporting Information).

**Fig. 2:**
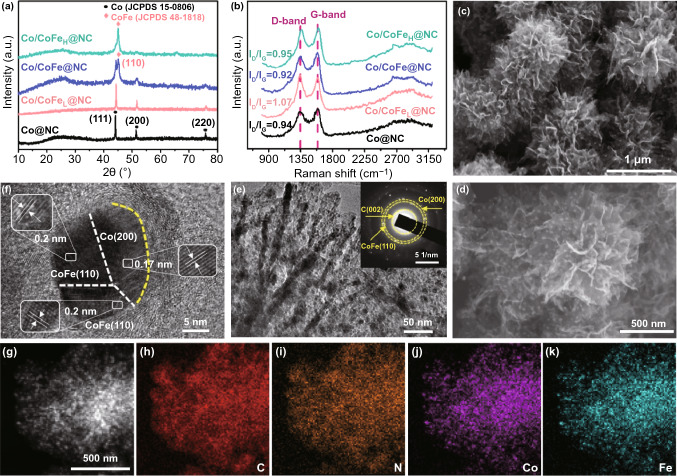
**a** XRD patterns, and **b** Raman spectra of various Co/CoFe_*x*_@NC samples with different Co/Fe ratios. **c, d** SEM images, and **e, f** TEM and HRTEM images of Co/CoFe@NC. **g-k** STEM-EDS elemental mapping images of C, N, Co and Fe atoms in Co/CoFe@NC

The crystal structure of the as-prepared materials was first characterized by XRD technology. The results (Fig. 2a) illustrate the phase transition from metallic Co to CoFe alloy by varying Fe proportion in the Co/Fe_*x*_-PPD precursor. With an appropriate amount of incorporated Fe, the XRD pattern confirms the formation of mixed crystal phases with metallic Co (JCPDS No. 15–0806) and face-centered cubic (*fcc*) CoFe alloy (JCPDS No. 48-1818) [4, 30]. The tunable composition of Co/CoFe_*x*_@NC makes it possible for optimizing the bifunctional electrocatalytic activities. As illustrated by Raman spectra in Fig. 2b, it is clear that two prominent peaks located at around 1335 and 1590 cm^−1^ can be observed for all samples, which are attributed to D-band and G-band derived from disordered carbon and the *E*_2g_ vibration of *sp*^2^-hybridized graphitic carbon. In general, the relative peak intensity ratio of D and G band (*I*_D_/*I*_G_) is used to evaluate the graphitization degree of carbonaceous materials [31]. The low *I*_D_/*I*_G_ ratio of Co/CoFe@NC (*I*_D_/*I*_G_ = 0.92) implies that the existence of Co and CoFe alloy can efficiently promote the formation of graphitic carbon, which is beneficial to improve the electrical conductivity and corrosion-resistance ability during electrocatalysis [9].

The morphology and microstructure of Co/CoFe@NC were examined by means of electron microscopies. As seen by SEM images in Fig. 2c, d, the Co/CoFe@NC preserves well the nanoflower morphology of Co-PPD precursor with an average size of 500 nm, which is assembled by dozens of 2D nanosheets. A great deal of well-dispersed nanoparticles with a size of approximate 10 nm are imbedded in the nanosheets. Similar observations are made on Co@NC, Co/CoFe_L_@NC, and Co/CoFe_H_@NC (Fig. S2), whereas Fe@NC displays nanowire morphology (Fig. S3). In Fig. 2e, the transmission electron microscopy (TEM) image of Co/CoFe@NC also reveals the presence of a large number of nanoparticles in nanosheets. The inset shows the selected-area-electron-diffraction (SAED) pattern, confirming the polycrystalline nature of the material as indicated by the discrete spots. Furthermore, the high-resolution TEM (HRTEM) image (Fig. 2f) demonstrates that the nanoparticles are surrounded by a few graphitic carbon layers. Such confinement effect not only prevents the Co/CoFe nanoparticles from agglomeration and detachment during the catalysis cycling, but also enriches the electron density on the carbon surface, thus inducing extra surface catalysis from carbon matrix (see theoretical calculation below). Interestingly, the Co/CoFe heterostructure is formed in a single nanoparticle as marked by the white lines. The lattice fringes with spacings of 0.17 and 0.20 nm can be readily assigned to the (200) plane of Co and the (110) plane of CoFe, respectively, which are consistent with the results of XRD [4, 30]. As reported, the heterostructure interfaces can enrich the catalytic active sites and promote the charge transfer between different components, thus enhancing the electrocatalytic performance [32–34]. The high-angle annular dark-field TEM (HAADF TEM) image and the corresponding element mappings (Fig. 2g–k) illustrate that the N, Co, and Fe elements are homogeneously dispersed throughout the entire carbon sheet.

**Fig. 3:**
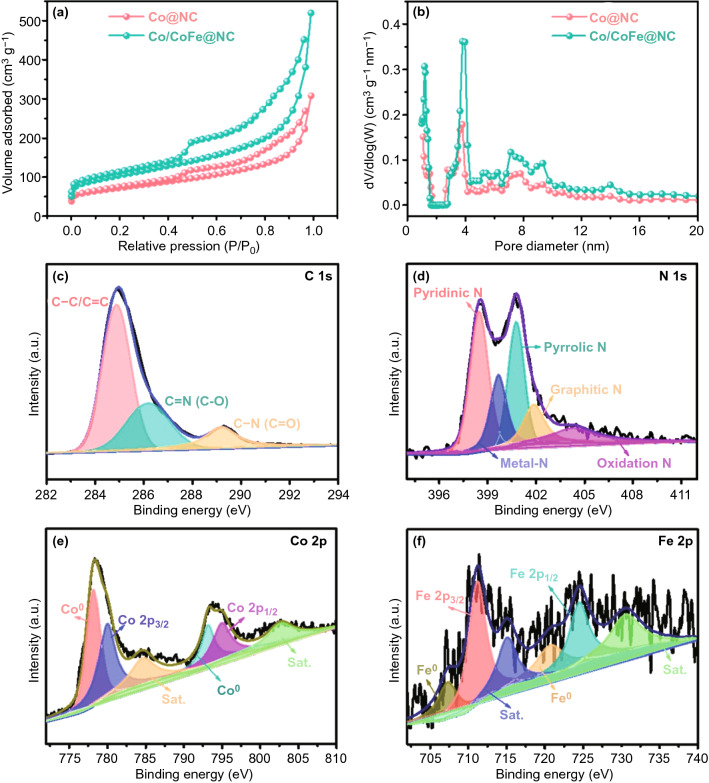
**a** Nitrogen adsorption–desorption isotherms, and **b** pore size distribution of Co@NC and Co/CoFe@NC. High-resolution XPS spectra of **c** C 1*s*, **d** N 1*s*, **e** Co 2*p* and **f** Fe 2*p* of Co/CoFe@NC

The specific surface area and porous structure were investigated by N_2_ adsorption/desorption isotherms. As shown in Fig. 3a, both Fe-free Co@NC and Co/CoFe@NC display typical-IV patterns with a distinct hysteresis loop at a higher N_2_ pressure (*P*/*P*_0_ = 0.4–1) according to the IUPAC classification, reflecting the unique mesoporous structure. In Fig. 3b, the pore size distribution also confirms the presence of mesopores [12]. The Brunaure–Emmett–Teller (BET) surface area of Co/CoFe@NC is calculated to be 468.6 m^2^ g^−1^, which is larger than that of Co@NC (255.4 m^2^ g^−1^). The large specific surface area is expected to provide more catalytic active sites with intimate catalyst/electrolyte contact.

X-ray photoelectron spectroscopy (XPS) measurements were performed to analyze surface elemental compositions and bonding configurations of Co/CoFe@NC. The survey XPS spectra in Fig. S4 affirms the existence of C, N, O, Co, and Fe elements and the atomic percentage of respective elements is listed in the inset table. The high-resolution C 1*s* XPS spectrum (Fig. 3c) can be deconvoluted into three subpeaks including C–C/C=C (284.6 eV), C=N/C–O (286.9 eV) and C–N/C = O (289.4 eV). The detected C-N component indicates that the N element has been successfully doped into the carbon lattice [7]. This is further demonstrated by N 1*s* core level spectrum in Fig. 3d. The subpeaks of N 1*s* XPS spectrum located at 398.5, 399.7, 400.3, 401.1, and 403.3 eV can be assigned to pyridinic-N, metal-N, pyrrolic-N, graphitic-N, and oxidized-N, respectively. The incorporation of N can modulate the charge distribution and spin density of the adjacent C atoms to produce Lewis base sites, thus enhancing the electrochemical performance [12]. In Fig. 3e, the Co 2*p* XPS spectrum exhibits two pairs of peaks. The first pair of peaks centered at 779.1 and 793.9 eV are assigned to zero-valence Co atom in metallic Co and CoFe alloy [35]. The other pair of peaks at 781.6 and 796.4 eV with shakeup satellites at 785.2 and 802.7 eV are ascribed to ionic-state peaks of Co, implying the formation of Co–N bonding. Similarly, the core level XPS of Fe 2*p* in Fig. 3f discloses the metallic state peaks at 720.1 and 709.2 eV and ionic-state peaks at 710.9 and 724.8 eV with shakeup satellite peaks at 714.4 and 732.5 eV, which are attributed to metallic Fe and Fe–N bonding, respectively [4]. The formation of Co–N and Fe–N bonding further demonstrates the strong interaction between Co/CoFe nanoparticles and N-doped carbon nanosheet, which can facilitate electron transfer and reduce interfacial resistance.

### Electrocatalytic Performance Toward OER

**Fig. 4:**
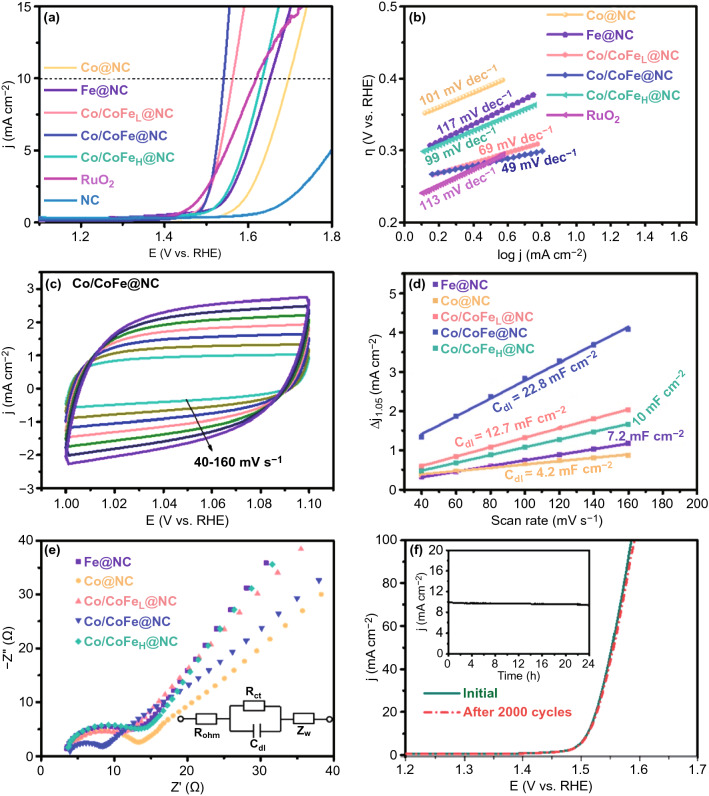
**a** OER polarization curves and **b** corresponding Tafel slopes of the as-prepared catalysts and RuO_2_ in 1 M KOH. **c** CV curves of Co/CoFe@NC in the double layer region at different scan rates. **d** Current density difference (Δj) at 1.05 V plotted against scan rates of various Co/CoFe_x_@NC samples. **e** EIS spectra recorded at a constant overpotential of 150 mV (inset: equivalent circuit, where *R*_ohm_ is ohmic resistance, *R*_ct_ is charge-transfer resistance, *C*_dl_ is interfacial capacitance and *Z*_w_ is warburg diffusion impedance). **f** Polarization curves for Co/CoFe@NC before and after 2000 CV cycles; the inset shows the long-term electrolysis curve of Co/CoFe@NC at an overpotential of 300 mV

The electrocatalytic OER performance of as-prepared samples and commercial RuO_2_ electrode were evaluated in 1 M KOH at a scan rate of 2 mV s^−1^. Figure 4a shows the linear sweep voltammetry (LSV) curves of various Co/CoFe_*x*_@NC electrocatalysts without *iR* correction. With the incorporation of Fe, the performance of Co/CoFe_*x*_@NC was significantly improved initially and then decreased. At an optimized Fe content, the Co/CoFe@NC achieves an OER current density of 10 mA cm^−2^ (*E*_*j*=10_) at the lowest overpotential of 300 mV, which is even superior to the benchmark RuO_2_ catalyst. The Tafel plots extracted from recorded LSV curves are used to probe the kinetics and intrinsic activities of electrocatalysts (Fig. 4b). Likewise, the Co/CoFe@NC exhibits the lowest Tafel slope of 49 mV dec^−1^, smaller than the benchmark RuO_2_ catalyst and other contrast samples. To reflect the performance difference more intuitively, the overpotentials at 10 mA cm^−2^ and Tafel slopes of as-prepared catalysts are summarized in Fig. S5, confirming further the favorable OER activity and reaction kinetics at the Co/CoFe@NC.

Electrochemical surface area (ECSA) and electrochemical impedance spectroscopy (EIS) were analyzed to further understand the partial underlying reason for the enhanced OER activity. ECSA was estimated based on the electrochemical double-layer capacitance (*C*_dl_) calculated from cyclic voltammetry (CV) at different scan rates (Figs. 4c and S6). As seen in Fig. 4d, *C*_dl_ value of Co/CoFe@NC is 22.8 mF cm^−2^, which is the largest among all examined samples. It indicates that the introduction of Fe can provide more electrochemically accessible active sites. The Nyquist plots (Fig. 4e) reveal that all catalysts have a semicircle at the high frequency region associated with charge transfer. Notably, the Co/CoFe@NC possesses the smallest semicircle diameter (*R*_ct_ = 8.9 Ω), confirming its faster charge transfer and lower electrode/electrolyte interfacial resistance [30, 36].

Aside from high OER activity, the long-term stability of Co/CoFe@NC was also examined. As displayed in Fig. 4f, after successive CV scans for 2000 cycles at a scan rate of 100 mV s^−1^, the polarization curve shows negligible difference as compared with the initial one. The inset of Fig. 4f shows the time-dependent current density curve of Co/CoFe@NC at a static potential of 1.5 V, and the material electrode maintains its catalytic activity for at least 20 h.

### Electrocatalytic Performance Toward ORR

**Fig. 5:**
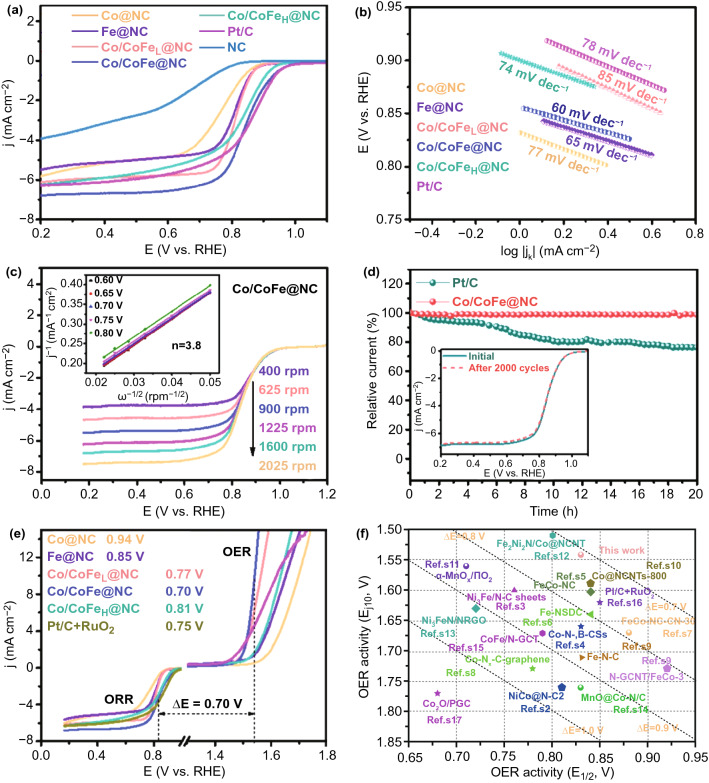
**a** ORR polarization curves and **b** corresponding Tafel slopes of the as-prepared catalysts and Pt/C in 0.1 M KOH. **c** RDE polarization profiles of Co/CoFe@NC at various rotation speeds and the inset of **c** shows the respective K-L plots for different voltages. **d** Chronoamperometric response of the Co/CoFe@NC and Pt/C at a constant potential of 0.6 V; the inset of **d** shows LSV curves of Co/CoFe@NC before and after chronoamperometric measurements. **e** Overall polarization curves of all samples over entire ORR and OER region. **f** Comparison of the bifunctional ORR (*E*_1/2_) and OER (*E*_*j*=10_) activities in this work with representative electrocatalysts reported recently (the dotted lines denote the ∆*E* at constant values)

To evaluate the bifunctionality of the as-prepared materials, their ORR performance was also investigated by a series of electrochemical measurements. Figure 5a demonstrates the representative LSV curves recorded by a rotating disk electrode (RDE) at 1600 rpm. As expected, the Co/CoFe@NC displays an impressive onset potential (*E*_onset_) of 0.97 V and a half-wave potential (*E*_1/2_) of 0.84 V, superior to that of Fe-free Co@NC catalyst (*E*_onset_ of 0.9 V and *E*_1/2_ of 0.75 V) and even comparable to that of Pt/C (*E*_onset_ of 0.98 V and *E*_1/2_ of 0.85 V). In addition, the Co/CoFe@NC exhibits the largest diffusion-limited current density of 6.8 mA cm^−2^, confirming further the beneficial effect of Fe-doping for ORR electrocatalysis. Figure 5b shows that the mass-transferred-corrected Tafel slope of Co/CoFe@NC (60 mV dec^−1^) is lower than that of Pt/C (78 mV dec^−1^), indicating its fast ORR kinetic process. Figures 5c and S7 reveal that the diffusion current densities increase with increasing the rotation speed from 400 to 2025 rpm, consistent with the accelerated diffusion of oxygen molecules from the electrolyte to the electrode surface [37]. Furthermore, the Koutecky–Levich (K-L) plots of Co/CoFe@NC with good linearity and the high coincidence at different potentials (inset of Fig. 5c) suggest the first-order reaction kinetics with respect to the concentration of dissolved oxygen [38]. The electron transfer number (*n*) derived from K-L plots is 3.8, implying that ORR on the Co/CoFe@NC dominantly follows an efficient four-electron transfer pathway.

To quantitatively evaluate the intermediate peroxide product, the rotating ring disk electrode (RRDE) measurements were also performed. As displayed in Fig. S8, the Co/CoFe@NC shows a high electron transfer number over 3.9 per O_2_ molecule and a HO_2_^‒^ yield below 5.0% in a wide potential range (0.2–0.8 V), both of which are superior to those of contrast catalysts and comparable to that of benchmark Pt/C. This result is in good agreement with the *n* value derived from the K-L plots. It verifies further that the highly efficient ORR on Co/CoFe@NC proceeds mainly via a dominant four-electron transfer pathway.

The Co/CoFe@NC catalyst also exhibits superior ORR stability, which is very important for the practical application of electrocatalysts. As shown in Fig. 5d, the relative activity of the Co/CoFe@NC catalyst remains 98% after 24 h of continuous operation at 0.65 V, far superior to 63% retention of Pt/C. The activity degradation of Pt/C could be at least partially attributed to the detachment of Pt nanoparticles from carbon supports, leading to agglomeration of nanoparticles, catalyst compaction and porosity loss [9]. In addition, LSV curves of Co/CoFe@NC before and after chronoamperometric measurements (Fig. 5d inset) remain quite consistent, suggesting further the excellent durability of Co/CoFe@NC. Because ORR is also an important half reaction in fuel cells, such as direct methanol fuel cell, the methanol crossover tolerance of Co/CoFe@NC was examined and compared with commercial Pt/C catalyst (Fig. S9). By spiking methanol into the electrolyte solution, the cathodic ORR current at Pt/C immediately changes to an anodic current owing to methanol oxidation, indicating severe disturbance of ORR by methanol crossover. By contrast, no significant current variation is observed for Co/CoFe@NC, suggesting the excellent methanol tolerance of Co/CoFe@NC.

The bifunctionality of oxygen electrocatalysts were assessed by the voltage difference (∆*E*) between *E*_1/2_ for ORR and the *E*_*j*=10_ for OER, in which the smaller ∆*E* indicates the limited electrochemical polarization and higher bifunctional activity. Remarkably, the Co/CoFe@NC affords the lowest ∆*E* of 0.70 V among various Co/CoFe_*x*_-based samples and noble-metal catalysts (Fig. 5e). Figure 5f summarizes the bifunctional electrocatalytic activity of as-prepared Co/CoFe@NC with those of recently reported composite catalysts based on transition metals (note detailed information in Table S1), corroborating an extraordinary reversible oxygen catalytic performance of Co/CoFe@NC.

### Theoretical Calculations and Mechanism Analysis

To further investigate and understand the intrinsically catalytic activity of Co/CoFe@NC, the adsorption Gibbs free energies (Δ*G*) of oxygen-containing intermediates were analyzed by density functional theory plus U (DFT + U) calculations. Because ORR on the Co/CoFe@NC follows a four-electron transfer pathway, the ORR and OER can be considered as reversible reactions. In alkaline condition, the widely accepted pathway for OER involves the following four elementary steps Eqs. 1–4, where * represents the active sites on catalysts, *OH, *O, and *OOH stand for the intermediate species adsorbed on the active sites, and ∆*G*_I_, ∆*G*_II_, ∆*G*_III_, and ∆G_IV_ represent for Gibbs free energies in each reaction. The overpotential η of the whole process is defined in Eq. 5 [18, 39, 40].

1 $$ {\text{OH}}^{ - } + * \,\rightarrow\, *{\text{OH}} + {\text{e}}^{ - } \quad \Delta G_{I} $$

2 $$ *{\text{OH}} + {\text{OH}}^{ - } \,\rightarrow\, *{\text{O}} + {\text{H}}_{2} {\text{O}} + {\text{e}}^{ - } \quad \Delta G_{II} $$

3 $$ *{\text{O}} + {\text{OH}}^{ - } \,\rightarrow\, *{\text{OOH}} + {\text{e}}^{ - } \quad \Delta G_{III} $$

4 $$ *{\text{OOH}} + {\text{OH}}^{ - } \,\rightarrow\, * + {\text{O}}_{2} + {\text{H}}_{2} {\text{O}} + {\text{e}}^{ - } \quad \Delta G_{IV} $$

5 $$ \eta = \max \,\left( {\Delta G_{I} , \, \Delta G_{II} ,\Delta G_{III} ,\Delta G_{IV} } \right)/{\text{e }} - \, 1.23\,{\text{V}} $$

**Fig. 6:**
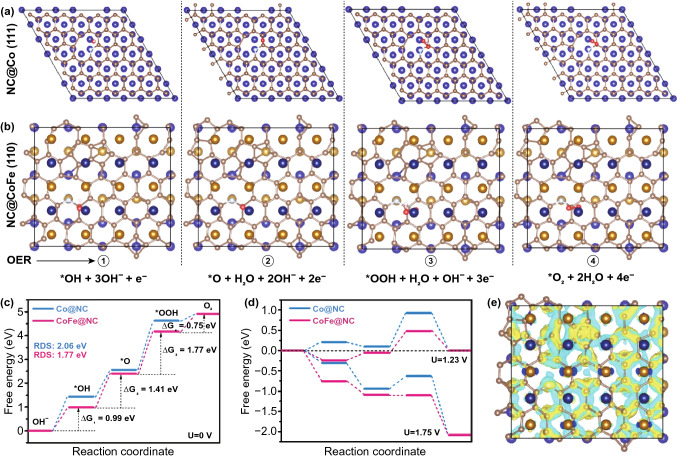
Optimized configurations of **a** Co@NC and **b** CoFe@NC with chemisorption of *OH, O*, *OOH and *O_2_ intermediates (atoms with yellow, blue, red, white and pink colors represent Fe, Co, O, H and N atoms, respectively). The calculated free energy diagrams for the OER on Co@NC and CoFe@NC at applied potentials of **c** 0 V, **d** 1.23 V (equilibrium potential) and 1.75 V. **e** Charge density difference of CoFe (110) layer and NC (111) layer (the yellow and blue isosurfaces show the electron gaining and losing, respectively)

The theoretical structure models of the OER intermediates adsorbed on the surfaces of Co@NC and CoFe@NC catalysts are presented in Figs. 6a, b and S10. When a potential of 0 V is applied, both catalysts display uphill pathways (Fig. 6c), where the step with maximum free energy change is the rate-determining step (RDS). The RDS at pristine Co@NC is found to be the formation of *OOH from *O group in step 3 with a high overpotential of 2.06 V. This is consistent with the result of earlier study that the catalyst can bind O too strongly and the overall reaction is limited by the formation of *OOH species [41, 42]. Interestingly, the binding strength of OER intermediates can be modulated by introducing alien elements, which promotes the activity of catalyst [43, 44]. By comparing the free energy profiles in Fig. 6c, CoFe@NC exhibits a lowered OER overpotential of 1.77 V for the RDS of *OOH formation step, confirming the improved OER performance after the incorporation of Fe atom into the Co@NC. On the other hand, the energy barrier for O_2_ desorption is altered in an opposite trend with the value increasing from 0.3 eV on Co@NC surface to 0.75 eV on CoFe@NC surface.

When the applied potential increases to 1.72 V that corresponds to a theoretical overpotential of 0.49 V, the highest ΔG value of the OER elementary steps (i.e., step 3) decreases to 0 eV. It suggests that the entire OER process can proceed spontaneously on the surface of CoFe@NC over this potential (Fig. 6d). For Co@NC, RDS is still hampered by an obvious energy barrier, requiring a higher applied potential to overcome. Thus, the comparison between Co@NC and CoFe@NC, from both theoretical and experimental analyses, points out the importance of engineering the Co/CoFe heterostructures for reducing the OER barrier and accelerating the reaction kinetic.

For the intimate contact between the supported metallic nanoparticles and the NC layer, the charge transfer between the two components was also simulated. As shown in Fig. 6e, electrons are transferred from the CoFe layer to the NC layer, causing an electron-rich state on the NC layer, which is favorable for the ORR/OER process by rapid electron release. The charge delocalization is also beneficial to the formation of abundant surface catalytic active centers [18]. The results of theoretical calculations corroborates further that Co/CoFe@NC possesses excellent oxygen electrocatalytic activity.

### Performance of Liquid and Quasi-Solid-State Zn–Air Batteries

**Fig. 7:**
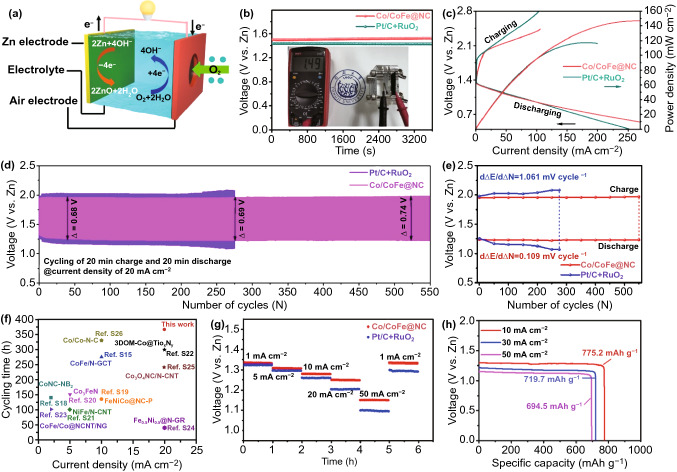
**a** Schematic diagram of a liquid ZAB. **b** Open-circuit plots, **c** galvanodynamic charge and discharge polarization curves and corresponding power density plots, **d** galvanostatic charge–discharge cycling profiles, and **e** voltage variation with cycles for ZABs assembled using Co/CoFe@NC and Pt/C + RuO_2_ catalysts, respectively. **f** Comparison of cyclability of ZABs between this work and other recently reported results. **g** Rate discharge curves and **h** specific capacity at various current densities

Considering the highly efficient bifunctional catalytic activity of Co/CoFe@NC, a homemade aqueous rechargeable ZAB was assembled with the catalyst-loaded carbon paper with gas diffusion layer as the air cathode and a polished zinc foil as the anode in electrolyte solution containing 6.0 M KOH and 0.2 M Zn(CH_3_COO)_2_ (Fig. 7a). For comparison, a battery employing a mixed composite of commercial Pt/C and RuO_2_ (with a 1:1 weight ratio) as an air electrode was also assembled. The Co/CoFe@NC-based battery affords a higher open-circuit voltage of 1.49 V than Pt/C + RuO_2_-based battery of 1.41 V (Fig. 7b). Figure 7c displays the galvanodynamic charge and discharge curves of the two air electrodes. A narrower voltage gap between the charge and discharge polarization voltages is found for Co/CoFe@NC, implying its better rechargeable capability than Pt/C + RuO_2_. Additionally, the Co/CoFe@NC air electrode delivers a higher peak power density (146.6 mW cm^−2^) than the noble-metal benchmark (117.3 mW cm^−2^), demonstrating further its superior catalytic activity.

The cycle stability and efficiency were evaluated by the galvanostatic charge–discharge mode at a high current density of 20 mA cm^−2^ for 40 min per cycle and the results are shown in Fig. 7d, e. The Co/CoFe@NC-based ZAB displays an initial charge–discharge voltage gap of 0.68 V, which is smaller than the ZAB assembled with Pt/C + RuO_2_ (0.87 V). More significantly, the ZAB based on Co/CoFe@NC exhibits only a very slight voltage loss of 60 mV after continuous test of 550 cycles (~ 360 h, 15 days), whereas the ZAB using Pt/C + RuO_2_ undergoes severe performance degradation with 292 mV increase in voltage gap after only 275 cycles (~ 180 h). It presents an extremely low decaying rate of 0.109 millivolts per cycle for the ZAB based on Co/CoFe@NC, which is ten times lower than the noble-metal benchmark (1.061 millivolts per cycle). The poor stability of the Pt/C + RuO_2_ electrode is presumably due to nanoparticle agglomeration and catalyst-support breakaway during the operation [45, 46]. For Co/CoFe@NC, the fascinating porosity and ultra-thin protective carbon shell can not only suppress the catalysts from agglomeration, but also maintain continuous electron/mass-transport channels for the ORR/OER. Such long-lasting cyclability over the time scale in this work is evidently superior to other recently reported results, as shown in Fig. 7f (note detailed information in Table S2).

The battery with Co/CoFe@NC also possesses good rate performance. As observed in Fig. 7g, the discharge plateau decreases with increasing the current density and the rate performance of the battery with Co/CoFe@NC is superior to that with Pt/C + RuO_2_ especially at the current density larger than 20 mA cm^−2^. Based on the mass of zinc, the specific capacities of our liquid ZAB are calculated to be 775.2, 719.7, and 694.5 mAh g^−1^ at current densities of 10, 30, and 50 mA cm^−2^, respectively (Fig. 7h). It is worth to note that the specific capacity of 775.5 mAh g^−1^ at 10 mA cm^−2^ is 94.5% of the theoretical capacity (820 mAh g^−1^) based on total Zn consumption.

**Fig. 8:**
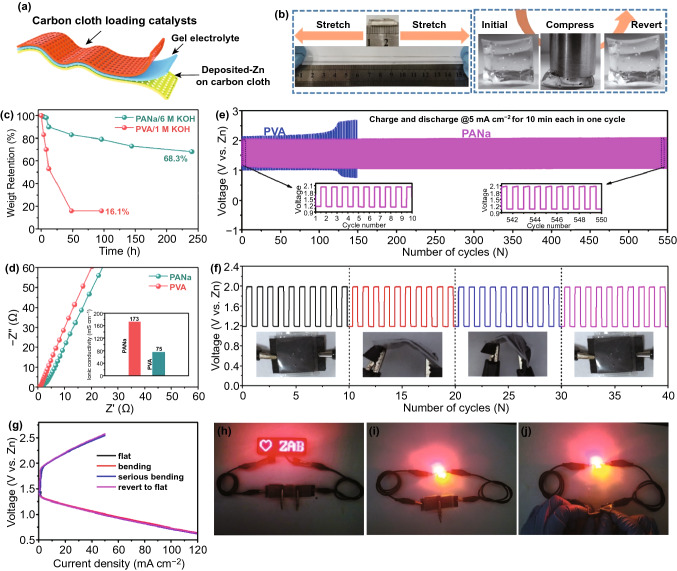
**a** Schematic illustration and demonstration of the quasi-solid-state flexible ZAB. **b** Optical pictures of PANa electrolyte in the original state, tensile state and compression state. **c** Comparison of water holding ability and **d** EIS with PANa and PVA electrolytes. **e** Long-term cycling performance by the use of PANa versus PVA electrolytes at a current density of 5 mA cm^−2^. **f** Cycling performances and **g** charge–discharge profile of the flexible ZAB under different bending states. **h-j** Optical pictures of a LED panel and three LED indicators powered by two flexible ZABs connected in series; the batteries in j are in the bending state

The Co/CoFe@NC catalyst was also assembled into a flexible quasi-solid-state ZAB to investigate its application for flexible devices. As illustrated in Fig. 8a, the catalyst-loaded carbon cloth serves as the air cathode, and the zinc-deposited carbon cloth (Fig. S11) is employed as the flexible anode. Furthermore, we prepared sodium polyacrylate hydrogel (PANa) with good alkaline-tolerance, stretchability, and water-retention capability as a promising quasi-solid-state electrolyte [47, 48] to overcome the disadvantages of polyvinyl alcohol (PVA) polymer electrolytes used commonly. Figures 8b and S12 show the mechanical property of the PANa hydrogel with 6 M KOH and 0.2 M Zn(CH_3_COO)_2_ intake, which is easily stretched, compressed, bended and twisted without any breakage or visible cracking, thus establishing its excellent mechanical property and alkaline tolerance. The PANa-based hydrogel also exhibits an excellent water intake capacity with 68.3% water retention after exposure in air over 10 days (Figs. 8c and S13). In contrast, the conventional PVA containing 1 M KOH begins to dry up after 12 h and only 16.1% water can be retained after exposure in air over 2 days. The excellent water holding ability of PANa-based electrolyte guarantees a larger ionic conductivity of 175 than 75 mS cm^−1^ for conventional PVA-based electrolyte (Fig. 8d).

As a result, the ZAB assembled with PANa-based hydrogel exhibits excellent cyclability. As shown in Fig. 8e, the initial charge and discharge voltages are 2.03 and 1.16 V, respectively, corresponding to a charge/discharge overpotential of 0.87 V with a round-trip efficiency of 57%. After 550 cycles over 92 h at 5 mA cm^−2^, the charge and discharge voltages only slightly change to 2.05 and 1.06 V, corresponding to a round-trip efficiency of 52%. In contrast, the ZAB assembled with PVA-based electrolyte shows severely deteriorated performance with the charge voltage increasing from 2.14 to 2.69 V and the discharge voltage decreasing from 1.01 to 0.76 V after only 150 cycles.

The flexible nature of the PANa-based electrolyte renders good flexibility to the ZAB as evidenced by the stable charge and discharge voltages under different bending states (Fig. 8f). The good flexibility of the ZAB is also proved by charge–discharge within a large current density range under different bending states (Fig. 8g). Finally, as a demonstration, two prototypical flexible ZABs connected in series can power the red LED screen of 2.5 V and simultaneously light up three LED indicators (Fig. 8h–j). These results clearly demonstrate the promising applications of our bifunctional electrocatalyst in a variety of wearable ZABs and other portable metal–air batteries.

## Conclusions

In summary, a strategy of coordination construction-cation exchange-pyrolysis is developed to fabricate heterostructured Co/CoFe nanoparticles embedded in N-doped graphitic carbon (NC) with a novel nanoflower structure. The synthesized catalyst delivers extraordinary ORR/OER bifunctional electrocatalytic performance with a small ∆*E* (*E*_*j*=10 _− *E*_1/2_) value of 0.70 V. Experimental and theoretical results collectively demonstrate the critical role of the heterointerface engineering of Co/CoFe nanoparticles and strong catalyst–support interaction in boosting the catalytic activity and stability. The assembled rechargeable ZAB employing the Co/CoFe@NC catalyst exhibits high specific capacity, low charge and discharge overpotential and exceptionally stable cyclability over 360 h (15 days), outperforming noble-metal benchmarks and other recently reported results. Furthermore, the Co/CoFe@NC and PANa hydrogel electrolyte are integrated into a flexible quasi-solid-state ZAB, demonstrating an excellent cycling performance and a good round-trip efficiency even under bending states. This study develops an excellent bifunctional oxygen electrocatalyst for ZABs by collective morphology-composition-structure engineering, and is significant for advanced flexible and wearable energy-storage devices.

## Supplementary Information

Below is the link to the electronic supplementary material.

Supplementary file 1 (PDF 1309 KB)
