# Supplementary material for: Engineering Two-Phase Bifunctional Oxygen Electrocatalysts with Tunable and Synergetic Components for Flexible Zn–Air Batteries
Source: Nanomicro Lett. 2021 May 15;13:126. doi: 10.1007/s40820-021-00650-2 (PMC8124028; doi:10.1007/s40820-021-00650-2)
Supplement: Supplementary file 1 — (PDF 1309 KB) [file 40820_2021_650_MOESM1_ESM.pdf]

Supporting Information for

Engineering Two-Phase Bifunctional Oxygen Electrocatalysts with Tunable and Synergetic Components for Flexible Zn-Air Batteries

Yanli Niu[1], Xue Teng[1], Shuaiqi Gong[1], Mingze Xu[1], Shi-Gang Sun[2,]*, Zuofeng Chen[1,]*

[1]Shanghai Key Laboratory of Chemical Assessment and Sustainability, School of Chemical Science and Engineering, Tongji University, Shanghai 200092, P. R. China

[2]State Key Lab of Physical Chemistry of Solid Surface, College of Chemistry and Chemical Engineering, Xiamen University, Xiamen 361005, P. R. China

*Corresponding authors. E-mail: sgsun@xmu.edu.cn (S. G. Sun); zfchen@tongji.edu.cn (Z. F. Chen)

S1 Experimental Section

Materials: Ruthenium oxide (RuO_2, ≥ 99.9%) and Nafion (5 wt % solution in aliphatic alcohols and water) were purchased from Sigma-Aldrich. Iron(III) nitrate nonahydrate ($Fe(NO_3)_3{\cdot}9H_2O$), cobaltous(II) nitrate hexahydrate ($(Co(NO_3)_2){\cdot}6H_2O$) and potassium hydroxide standard solution (1 M, 99%) were obtained from Aladdin Reagent. Methanol (CH_3OH, ≥ 99.9%), *p*-phenylenediamine ($C_6H_4(NH_2)_2$, PPD), *n*-hexane (C_6H_{14}) and zinc acetate ($Zn(CH_3COO)_2$) were bought from Shanghai Macklin Chemical Reagent Co. Ltd. Commercial 20 wt% Pt/C was purchased from Johnson-Matthey. All chemical reagents in this work were analytical grade and employed without further purification. Deionized water (18.0 MΩ·cm) was used throughout all experiments.

Synthesis of Co-PPD complex: In a typical procedure, 1.0 g *p*-phenylenediamine was dissolved in 20 mL methanol, which was subsequently added into 20 mL methanol containing 0.9 g $Co(NO_3)_2){\cdot}6H_2O$. After stirring for a while, the homogeneous solution was transferred into a 50 mL Teflon-lined autoclave, which was sealed and maintained at 120 °C for 8 h and then naturally cooled to room temperature. The resulting precipitate was collected by centrifuge and washed with methanol and ethanol, followed by vacuum drying at 60 °C overnight.

Synthesis of Fe-PPD complex: The Fe-PPD complex was also prepared by similar procedures by using $Fe(NO_3)_3{\cdot}9H_2O$ to substitute $Co(NO_3)_2){\cdot}6H_2O$.

Synthesis of Co/Fe$_x$-PPD complex: Typically, 50 mg of as-prepared Co-PPD complex was dispersed in 12 mL of *n*-hexane by sonication for 1 h to obtain a homogeneous suspension. Afterwards, 280 μL of $Fe(NO_3)_3{\cdot}9H_2O$ methanol solution (25 mg mL^{-1}) was added dropwise to the above solution and then the mixed solution was kept for another 1 h under continuous magnetic stirring at room temperature. In

this process, protons produced from the slow hydrolysis of Fe^{3+} can induce partial Co^{2+} to be dissociated from Co-PPD, which is accompanied with Fe^{3+} coordination to the organic ligand as nodes. This cation exchange process is evidenced by supernatant in pink color, caused by Co^{2+} released during the reaction. Finally, the resulting product (Co/Fe-PPD) was collected by centrifugation, washed with ethanol for several times and dried at 60 °C overnight. We also prepared a couple of samples by varying Fe^{3+} contents introduced into the Co-PPD complex. The products were denoted as Co/Fe_L-PPD and Co/Fe_H-PPD, respectively, where subscript L and H represent a low (70 μL) and high (420 μL) amounts of added Fe^{3+}. In general, these Fe^{3+}-incorporated samples (Co/Fe-PPD, Co/Fe_L-PPD and Co/Fe_H-PPD) maintained the nanoflower structure of Co-PPD. However, when the amount of added Fe^{3+} was over 700 uL, the nanoflower structure of the resultant sample would be destroyed, tending to form featureless nanospheres.

Synthesis of the Co/CoFe$_x$@NC catalyst: The Co/CoFe$_x$@NC catalyst was synthesized by pyrolyzing the Co/Fe$_x$-PPD complex in a tube furnace under an Ar atmosphere at 800 °C for 2 h with a ramp rate of 5 °C/min. In the paper, the Co/CoFe@NC signifies a sample with optimized content of Fe, while the Co/CoFe$_L$@NC and Co/CoFe$_H$@NC denote samples with low and high contents of Fe, respectively.

Monometallic Co@NC and Fe@NC catalysts were synthesized by pyrolyzing the Co-PPD and Fe-PPD complex in a tube furnace under an Ar atmosphere at 800 °C for 2 h with a ramp rate of 5°C/min, respectively.

Physical characterization: The structures and compositions of samples were analyzed by powder X-ray diffraction (XRD) using an X-ray diffractometer (Bruker Foucs D8) with Cu Kα radiation (λ=1.54178 Å, 40 kV and 40 mA). Fourier transform infrared (FTIR) spectra were recorded from KBr disks using a Thermo Scientific Nicolet 6700 spectroscopy instrument with a scan range of 400-4000 cm^{-1}. The scanning electron microscopy (SEM) images were collected on a Hitachi S-4800 (Hitachi, Japan). The transmission electron microscopy (TEM) images, high resolution transmission electron microscopy (HRTEM) images, the selected-area-electron-diffraction (SAED) pattern and energy-dispersive X-ray spectroscopy (EDS) elemental mapping images were collected on a microscope (JEM2100F, JEOL, Japan) with an accelerating voltage of 200 kV. The Brunauer-Emmett-Teller (BET) method and the Barrett-Joyner-Halenda (BJH) model were utilized to calculate the specific surface areas and pore size distributions, respectively. The chemical state and elemental composition of samples were analyzed using X-ray photoelectron spectroscopy (XPS ESCALAB 250) technique, all data were corrected using the C 1s peak at 284.8 eV as an internal standard. Raman spectra were recorded on a confocal microscope laser Raman spectrometer (Rainshaw invia).

Density functional theory (DFT) calculation details: Calculations were carried out with DFT implanted in the Vienna Ab-initio Simulation Package (VASP) to give a better understanding for the superior activity of Co/CoFe@NC. The Perdew-Burke-

Ernzerhof (PBE) parametrization of the generalized gradient approximation (GGA) is for the exchange-correlation functional. The energy cut-off (ECUT) of the plane wave basis was set as 500 eV and a gamma centered 7*7*3 Monkhorst-Pack k-point (KPOINTS) mesh was applied for the k-point samples in the Brillouin zone for the surface model geometry optimization. The tolerance of electronic and ionic relaxation is 10^{-5} eV and 0.01 eV/A respectively.

Electrochemical measurements: The electrocatalytic activities of ORR and OER were evaluated by employing a CHI 760E electrochemical workstation (Shanghai Chenhua instrument, China) coupled with a Pine rotator in a standard three-electrode system. For a working electrode, 5 mg of catalyst was ultrasonically dispersed in a mixed solution containing 0.49 mL of isopropanol and 10 μL of 5 wt% Nafion solution to form a homogeneous suspension. The 10 μL of the as-prepared catalyst ink was evenly dropped on a clean rotating disk working electrode (RDE) or rotating ring-disk electrode (RRDE) with a catalyst loading of 0.25 mg cm^{-2}. The Hg/HgO (1 M KOH) and carbon rod electrodes were used as reference and counter electrodes, respectively and all measured potentials (*vs.* Hg/HgO) in this work were converted to a RHE scale via calibrated equation $E_{RHE} = E_{\mathrm{Hg/HgO}} + 0.059pH + 0.098$.

ORR measurements: All the measurements were carried out in O_2-saturated 0.1 M KOH solution. Linear sweep voltammetry (LSV) tests were conducted at a scan rate of 5 mV s^{-1} with different rotation speeds (400, 625, 900, 1225, 1600 and 2025 rpm) from 0.2 to 1.05 V versus RHE. Koutecky-Levich plots (J^{-1} *vs.* $\omega^{-1/2}$) were fitted into the linear curves, where the slopes and intercepts can be used to calculate the electron transfer number (n) and kinetic current density (J_k) number according to the Koutecky-Levich equation:

$$J = \frac{1}{J_K} + \frac{1}{J_L} = \frac{1}{J_K} + \frac{1}{B\,\omega^{-1/2}}$$

$$\mathrm{B} = 0.2\mathrm{nF}C_0 {D_0}^{2/3} v^{-1/6}$$

$$J_k = nFkC_0$$

Where J is the measured current densities (mA cm^{-2}), J_K is the kinetics current density (mA cm^{-2}) and J_L is the diffusion-limiting current densities (mA cm^{-2}), ω is the rotating speed (rpm), F is the Faraday constant (96485 C mol^{-1}), C_0 is the bulk concentration of O_2 (1.2×10^{-6} mol cm^{-3}), ν is the kinematic viscosity of the electrolyte (0.01 cm^2 s^{-1}), D_0 is the O_2 diffusion coefficient (1.9×10^{-5} cm^2 s^{-1}), and k is the electron-transfer rate constant.

The electron transfer number (n) and the yield of hydrogen peroxide (H_2O_2%) can also be calculated based on the rotating ring-disk electrode (RRDE) measurements using the following equation:

$$n = 4 \times \frac{I_d}{I_r/N + I_d}$$

$$H_2O_2\ (\%) = 200 \times \frac{I_r/N}{I_r/N + I_d}$$

Where I_d represents the disk current and I_r represents the ring current. N is the current collection efficiency of the Pt ring which was determined to be 0.37. The ring potential is fixed at 1.5 V *vs.* RHE.

OER measurements: Prior to measurements, cyclic voltammetry (CV) scanning was conducted at a scan rate of 50 mV s^{-1} for 100 cycles to activate the catalyst in N_2-saturated 1 M KOH electrolyte. After that, the electrochemically active surface areas (ECSA) of the samples were roughly evaluated based on the electrochemical double-layer capacitance (C_{dl}), which was determined from CV curves recorded at different scan rates from 40 to 160 mV s^{-1} in the non-Faradaic potential region (1.0-1.1 V). The value of C_{dl} was calculated according to the following equation:

$$C_{dl} = \frac{j_a - j_c}{2v} = \frac{\Delta j}{2v}$$

Where Δj is the difference between anodic and cathodic current densities recorded at the middle of selected potential range, and v is the scan rate.

The OER polarization curves were measured at a potential window of 0 - 1 V (*vs.* RHE) with a sweep rate of 2 mV s^{-1} and all the LSV curves were presented without any iR correction. Tafel slopes were calculated from the LSV curves by plotting overpotential (η) against log (j). The electrochemical impedance spectroscopy (EIS) measurements were carried out at various overpotentials from 10 kHz to 0.1 Hz with an amplitude of 5 mV. The stability of optimized catalyst was characterized by chronopotentiometric test with a constant current at 10 mA cm^{-2} for 12 h.

Assembly and test of aqueous Zn-air batteries: A home-made aqueous Zn-air battery was assembled with the optimized catalyst loaded carbon paper with gas diffusion layer as the air cathode (mass loading: 1 mg cm^{-2}), a polished Zn foil (thickness: 0.3 mm) as the anode, and 6 M KOH + 0.2 M Zn $(CH_3COO)_2$ as the electrolyte. For comparison, commercial Pt/C and RuO_2 mixed catalyst (mass ratio of 1:1) was also prepared by the same procedure to fabricate a liquid primary Zn-air battery. All tests were operated under an ambient atmosphere (oxygen from air). The polarization curves were recorded using a CHI 760E electrochemical working station with the open circuit potential as the initial potential at a scan rate of 2 mV s^{-1} and both the current density and power density were normalized to the geometric surface area of the cathode. The galvanostatic discharge-charge curves were collected on a LAND testing station at a current density of 20 mA cm^{-2} with 40 min per cycle (20 min for charging and 20 min for discharging).

Assembly and test of flexible quasi-solid-state Zn-air batteries: The home-made

flexible quasi-solid-state Zn-air battery was fabricated by using zinc-deposited carbon cloth as the anode, and the as-prepared catalyst loaded on a hydrophilic carbon cloth (0.5×2.0 cm^{-2}, catalyst loading: 1 mg cm^{-2}) as the air cathode. The hydrogel polymer electrolyte was prepared as follows:[1] A concentrated sodium hydroxide solution (5 mL, 20 M) was slowly dropped into an aqueous solution of acrylic acid monomer (7.2 mL, 47 wt%) under vigorous stirring. Thereafter, 110 mg of ammonium persulfate and 4 mg of N, N′-methylenebis-acrylamide were added to the above mixture solution followed by stirring for 30 min at room temperature. Before polymerization, the above mixture solution was degassed with N_2. Subsequently, the free-radical polymerization was initiated to proceed at 45 °C for 30 h. Finally, the as-prepared polymer was peeled off and fully dried in an 80 °C oven and then soaked in a mixed solution containing 6 M potassium hydroxide. The flexible quasi-solid-state Zn-air battery was assembled by attaching as-prepared air cathode and Zn anode on the two sides of gel electrolyte. The cycling test of Zn-air battery was conducted using a recurrent galvanostatic pulse method for 5 min of discharge followed by 5 min of charge at a current density of 5 mA cm^{-2}. The power density of Zn-air battery was calculated by $P = IV$, where I is the discharge current density and V is the corresponding voltage. The specific capacity was calculated according the following equation:

$$\text{Specific capacity} = \frac{\text{current} * \text{service hours}}{\text{weight of consumed zinc}}$$

The following equation is used to calculate hydrogel electrolyte ionic conductivities (σ):

$$\sigma = d/RS$$

Where d represents the thickness of electrolyte, S is the area of the electrolyte, and R is the ohmic resistance obtained from the electrochemical impedance spectrum.

S2 Supplementary Tables and Figures

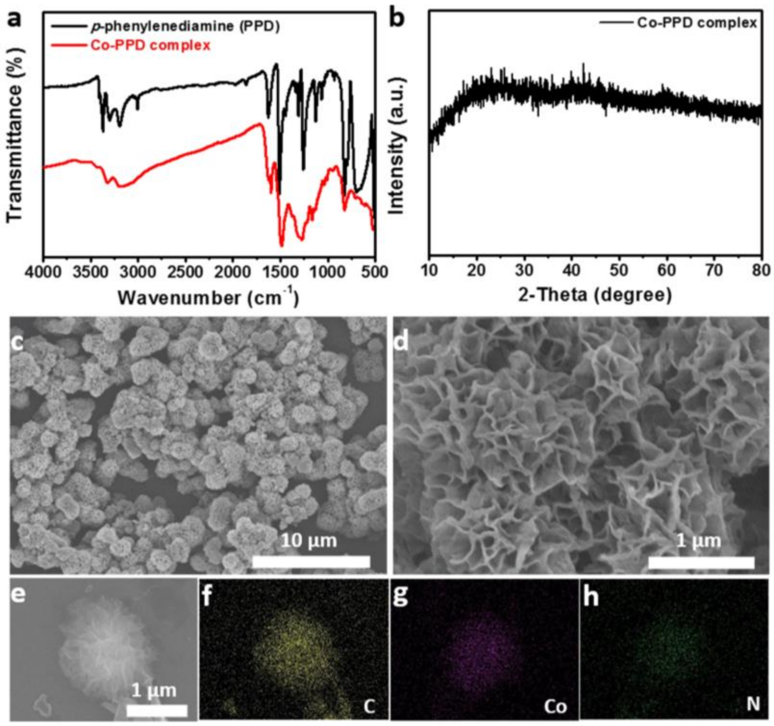

Fig. S1 a FTIR spectra of *p*-phenylenediamine and Co-PPD complex. **b** XRD pattern, **c-e** SEM images and **f-h** corresponding EDS elemental mapping images of C, Co, and N elements of Co-PPD complex

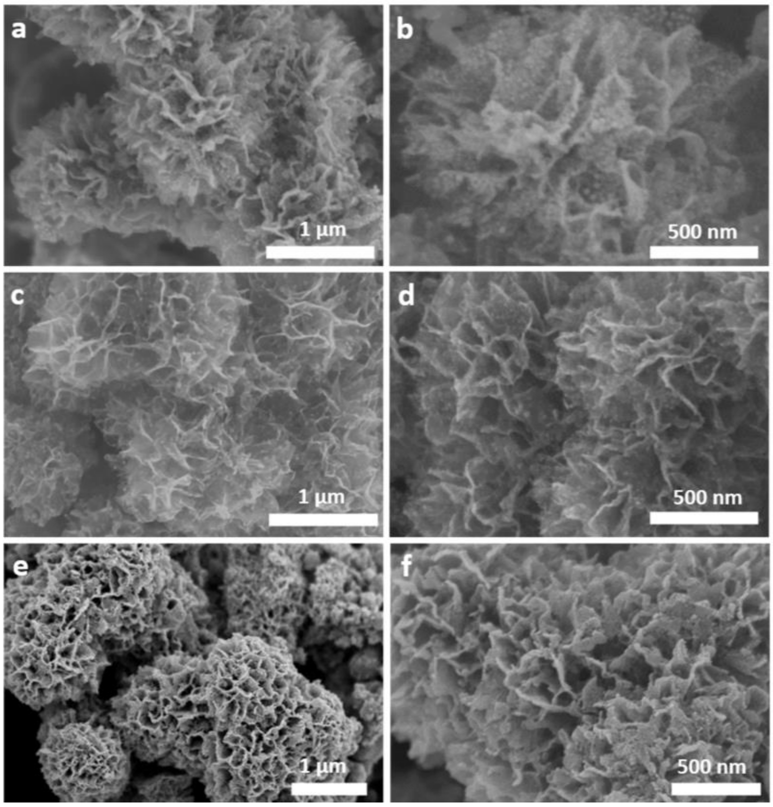

Fig. S2 SEM images of **a, b** Co@NC, **c, d** Co/CoFe$_L$@NC, and **e, f** Co/CoFe$_H$@NC catalysts

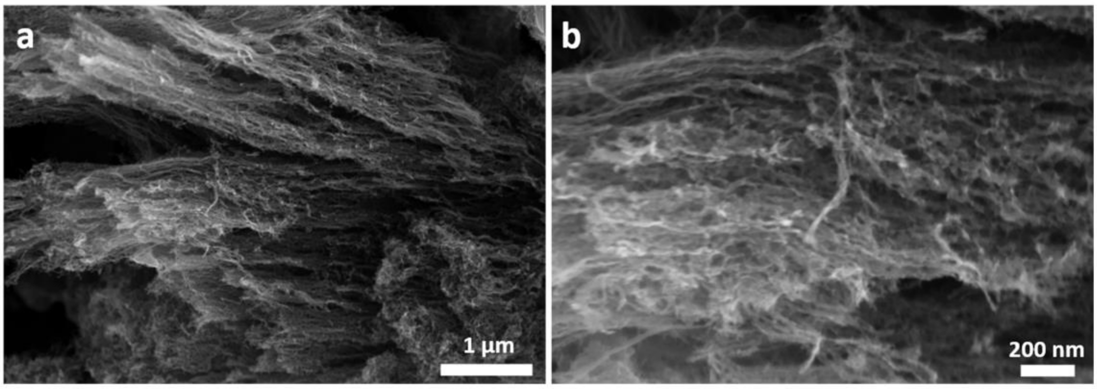

Fig. S3 SEM images of Fe@NC at **a** low and **b** high magnifications

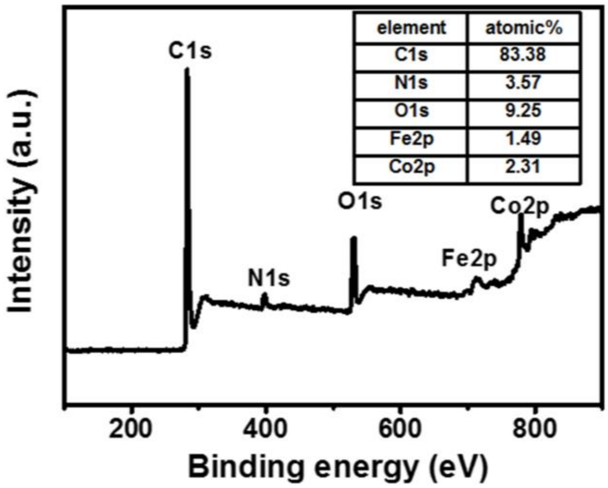

Fig. S4 XPS survey spectrum of as-prepared Co/CoFe@NC catalyst

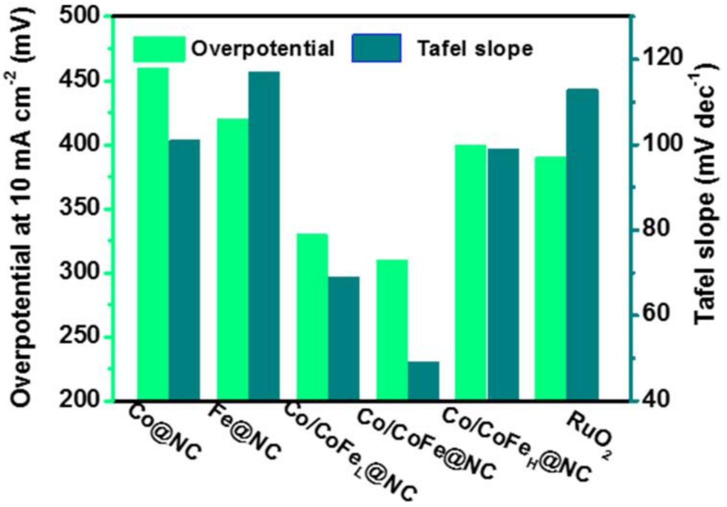

Fig. S5 Comparison of overpotentials and Tafel slopes of different electrodes and RuO_2 toward OER

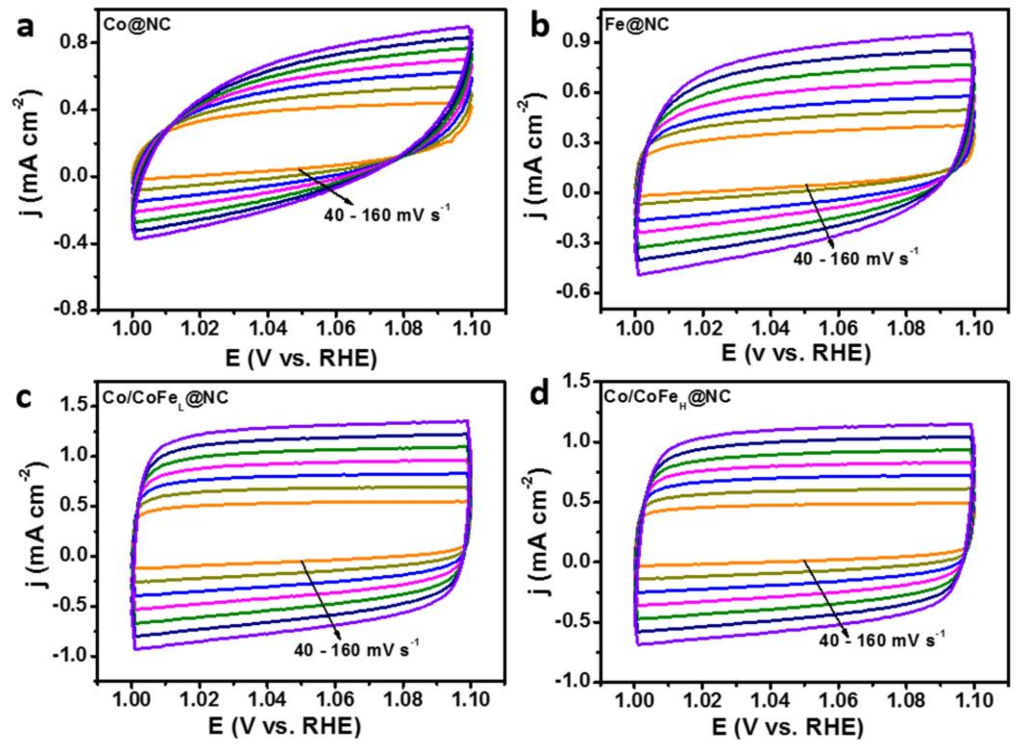

Fig. S6 CV curves in the double layer region at different scan rates for **a** Co@NC, **b** Co/CoFe$_L$@NC, **c** Fe@NC and **d** Co/CoFe$_H$@NC

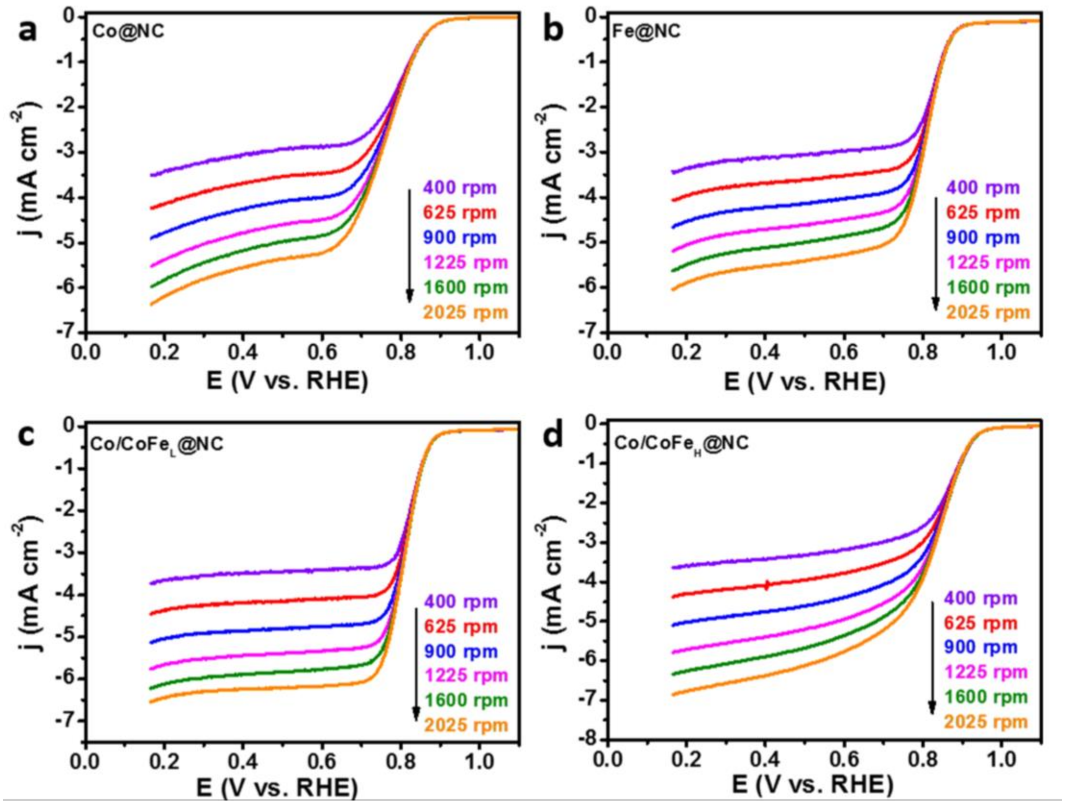

Fig. S7 RDE polarization profiles of **a** Co@NC, **b** Fe@NC, **c** Co/CoFe$_L$@NC and **d** Co/CoFe$_H$@NC at various rotation speeds

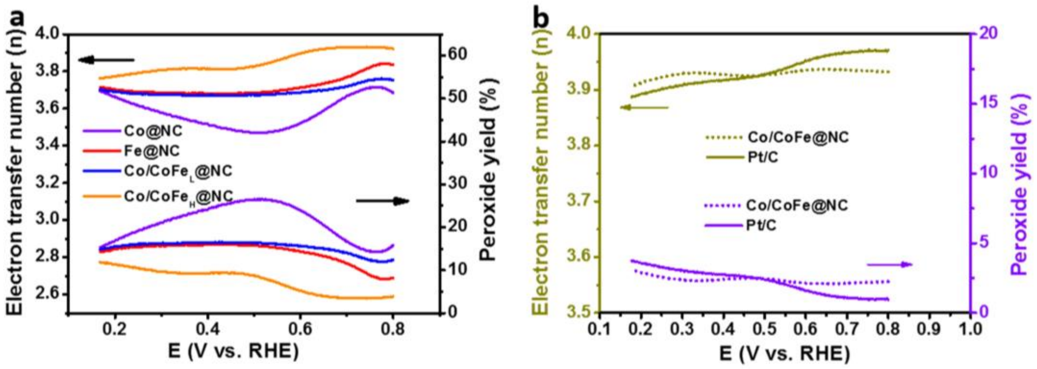

Fig. S8 RRDE-calculated electron transfer numbers and HO_2^- yield during ORR for different catalysts and Pt/C

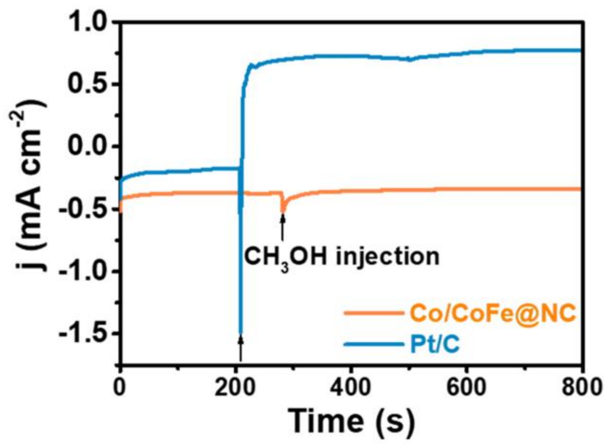

Fig. S9 Amperometric curves of Co/CoFe@NC and commercial Pt/C at 0.65 V in O_2-saturated 0.1 M KOH solution. The arrows indicate the addition of 2% (v/v) methanol into the electrolyte solution

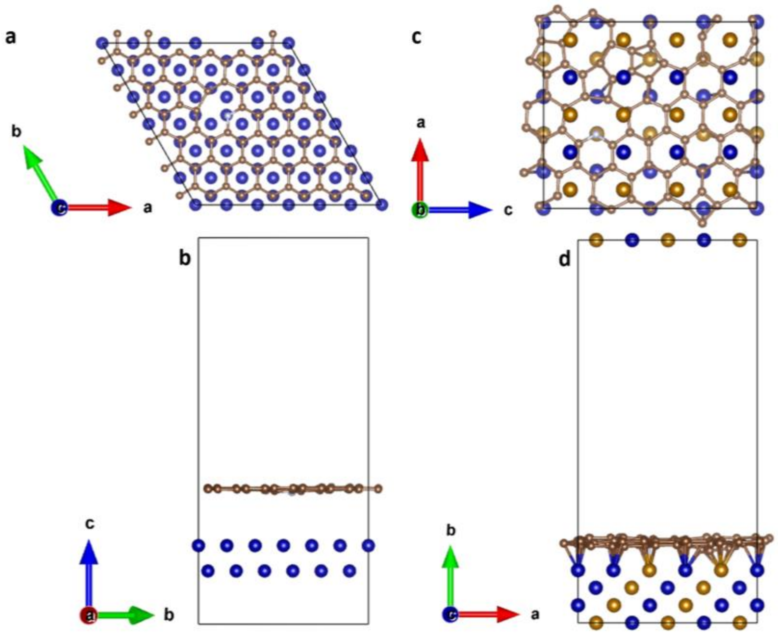

Fig. S10 Corresponding structural models of **a, b** Co@NC and **c, d** CoFe@NC

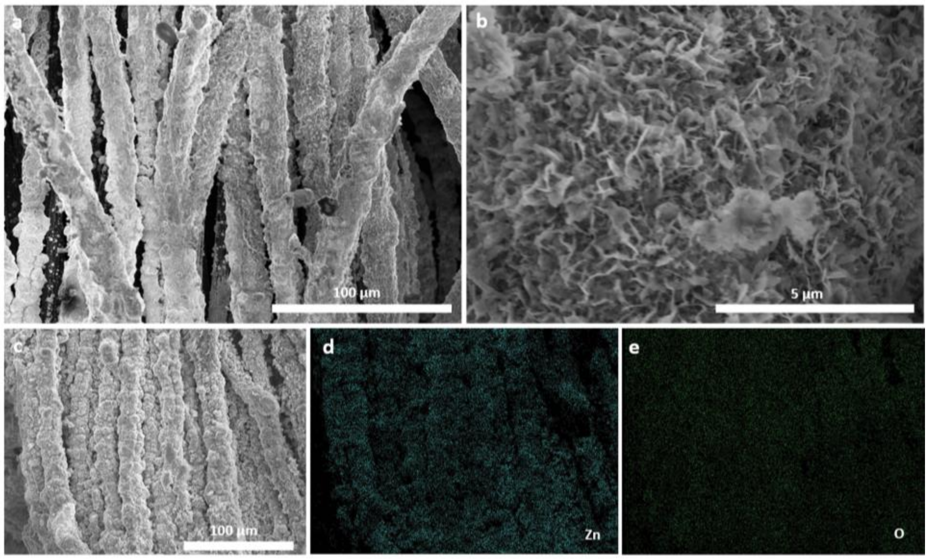

Fig. S11 a-c SEM images of deposited metallic Zn on carbon cloth and corresponding EDS mapping images of **d** Zn and **e** O elements

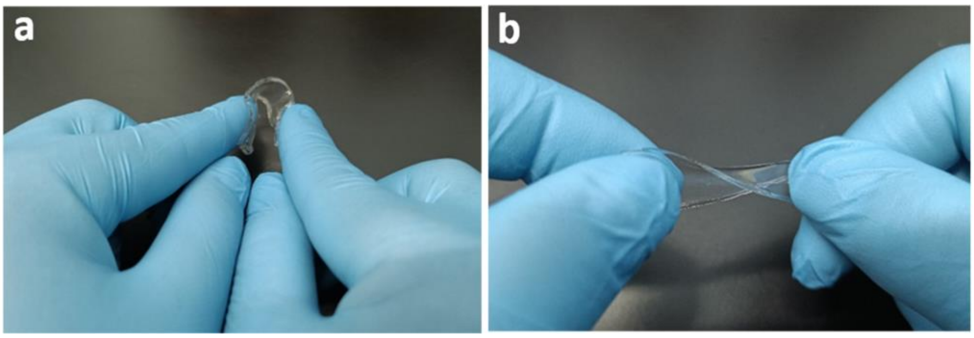

Fig. S12 Optical photos of the PANa-based hydrogels at **a** bending and **b** twisty states

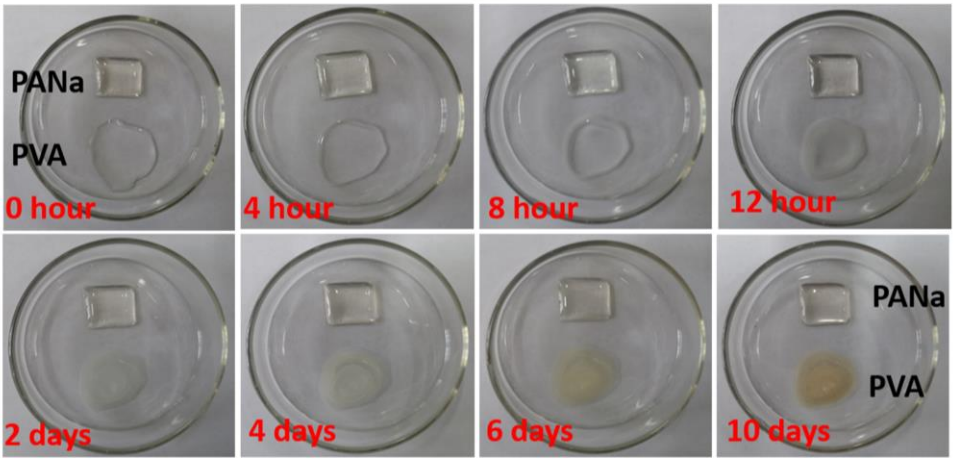

Fig. S13 Comparison of water holding ability of PANa/6 M KOH and PVA/1 M KOH basic hydrogels

Table S1 Comparison of the ORR/OER activities of Co/CoFe@NC catalyst with recently reported bifunctional electrocatalysts

Catalysts	ORR: $E_{1/2}$ (V)	OER: $E_{j=10}$ (V)	$\Delta E = E_{j=10} - E_{1/2}$ (V)	Refs.
**Co/CoFe@NC**	**0.84**	**1.54**	**0.70**	**This work**
N-GCNT/FeCo-3	0.92	1.73	0.81	[S2]
NiCo@N-C2	0.81	1.76	0.95	[S3]
Ni_3Fe/N-C sheet	0.76	1.60	0.84	[S4]
Co-N,B-CSs	0.83	1.66	0.83	[S5]
FeCo-NCps	0.84	1.60	0.76	[S6]
Fe-NSDC	0.84	1.64	0.80	[S7]
FeCo-N_x-CN-30	0.88	1.67	0.79	[S8]
Co-N_x-C-graphene	0.78	1.73	0.95	[S9]
Fe-N-C	0.83	1.71	0.88	[S10]
Co@NCNTs-800	0.84	1.59	0.75	[S11]
α-MnOx/TiO_2	0.71	1.56	0.76	[S12]
Fe_2Ni_2N/Co@NCNT	0.80	1.51	0.71	[S13]
Ni_3FeN/NRGO	0.72	1.63	0.91	[S14]
MnO@Co-N/N	0.83	1.76	0.93	[S15]
CoFe/N-GCT	0.79	1.67	0.88	[S16]
Pt/C+RuO_2	0.85	1.62	0.77	[S17]
Co_3O_4/PGC	0.68	1.77	1.09	[S18]

Note: ORR and OER measurements were carried out in 0.1 M and 1 M KOH electrolyte, respectively.

Table S2 Performance of liquid rechargeable Zn-air batteries assembled with Co/CoFe@NC catalyst and other bifunctional oxygen electrocatalysts reported in literature

Catalysts	Peak power density ($mW\ cm^{-2}$)	Charge/ discharge voltage gap (V)	Specific capacity ($mAh\ g_{Zn}^{-1}$)	Stability	Refs.
**Co/CoFe@NC**	**146.6**	**0.68@10 mA cm^{-2}**	**775.2@5 mA cm^{-2}**	**Life time of over 360 h @20 mA cm^{-2}**	**This work**
CoNC-NB2	104	0.92@25 mA cm^{-2}	698@5 mA cm^{-2}	Life time of over 140 h @2 mA cm^{-2}	[S19]
FeNiCo@NC-P	112	0.84@10 mA cm^{-2}	N/A	Life time of over 135 h @10 mA cm^{-2}	[S20]
CoFe/N-GCT	203	0.80@10 mA cm^{-2}	748@20 mA cm^{-2}	Life time of over 275 h @10 mA cm^{-2}	[S16]
Co_3FeN	108	0.90@5 mA cm^{-2}	890@5 mA cm^{-2}	Life time of over 150 h @5 mA cm^{-2}	[S21]
NiFe/N-CNT	300.7	0.72@10 mA cm^{-2}	772@10 mA cm^{-2}	Life time of over 100 h @5 mA cm^{-2}	[S22]
3DOM-Co@TiO_xN_y	110	0.97@10 mA cm^{-2}	697@20 mA cm^{-2}	Life time of over 300 h @20 mA cm^{-2}	[S23]
CoFe/Co@NC NT@NG	161	0.82@20 mA cm^{-2}	N/A	Life time of over 100 h @2 mA cm^{-2}	[S24]
Fe0.5Ni0.5@N -GR	85	0.82@20 mA cm^{-2}	765@10 mA cm^{-2}	Life time of over 40 h @20 mA cm^{-2}	[S25]
Co_3O_4NC/N-CNT	92	1.02@20 mA cm^{-2}	N/A	Life time of over 240 h @20 mA cm^{-2}	[S26]
Co/Co-N-C	132	0.82@10 mA cm^{-2}	N/A	Life time of over 330 h @10 mA cm^{-2}	[S27]

Supplementary References

[S1] Y. Huang, Z. Li, Z. Pei, Z. Liu, H. Li et al., Solid-state rechargeable Zn//NiCo and Zn-air batteries with ultralong lifetime and high capacity: the role of a sodium polyacrylate hydrogel electrolyte. Adv. Energy Mater. **8**, 1802288 (2018). https://doi.org/10.1002/aenm.201802288

[S2] C.-Y. Su, H. Cheng, W. Li, Z.-Q. Liu et al., Atomic modulation of FeCo-nitrogen-carbon bifunctional oxygen electrodes for rechargeable and flexible all-solid-state Zinc-air battery. Adv. Energy Mater. **7**, 1602420 (2017). https://doi.org/10.1002/aenm.201602420

[S3] Y. Fu, H.-Y. Yu, C. Jiang, T.-H. Zhang, R. Zhan et al., NiCo alloy nanoparticles decorated on N-doped carbon nanofibers as highly active and durable oxygen electrocatalyst. Adv. Funct. Mater. **28**, 1705094 (2018). https://doi.org/10.1002/adfm.201705094

[S4] G. Fu, Z. Cui, Y. Chen, Y. Li, Y. Tang et al., Ni_3Fe-N doped carbon sheets as a bifunctional electrocatalyst for air cathodes. Adv. Energy Mater. **7**, 1601172 (2017). https://doi.org/10.1002/aenm.201601172

[S5] Y. Guo, P. Yuan, J. Zhang, Y. Hu, I. S. Amiinu et al., Carbon nanosheets containing discrete Co-N_x-B_y-C active sites for efficient oxygen electrocatalysis and rechargeable Zn-air batteries. ACS Nano **12**, 1894-1901 (2018). https://doi.org/10.1021/acsnano.7b08721

[S6] J. Liu, T. He, Q. Wang, Z. Zhou, Y. Zhang et al., Confining ultrasmall bimetallic alloys in porous N–carbon for use as scalable and sustainable electrocatalysts for rechargeable Zn–air batteries. J. Mater. Chem. **7**, 12451-12456 (2019). https://doi.org/10.1039/C9TA02264C

[S7] J. Zhang, M. Zhang, Y. Zeng, J. Chen, L. Qiu et al., Single Fe atom on hierarchically porous S, N-codoped nanocarbon derived from porphyra enable boosted oxygen catalysis for rechargeable Zn-air batteries. Small **15**, 1900307 (2019). https://doi.org/10.1002/smll.201900307

[S8] S. Li, C. Cheng, X. Zhao, J. Schmidt, A. Thomas, Active salt/silica-templated 2Dmesoporous FeCo-N_x-Carbon as Bifunctional oxygen electrodes for Zinc–air Batteries. Angew. Chem. Int. Ed. **130**, 1856-1862 (2018). https://doi.org/10.1002/anie.201710852

[S9] C. Tang, B. Wang, H. F. Wang, Q. Zhang, Defect engineering toward atomic Co–N_x–C in hierarchical graphene for rechargeable flexible solid Zn-air batteries. Adv. Mater. **29**, 1703185 (2017). https://doi.org/10.1002/adma.201703185

[S10] J. Wang, H. Wu, D. Gao, S. Miao, G. Wang et al., High-density iron nanoparticles encapsulated within nitrogen-doped carbon nanoshell as efficient oxygen electrocatalyst for Zinc–air battery. Nano Energy **13**, 387-396 (2015). https://doi.org/10.1016/j.nanoen.2015.02.025

[S11] Q. Wang, K. Ye, L. Xu, W. Hu, Y. Lei et al., Carbon nanotube-encapsulated cobalt for oxygen reduction: integration of space confinement and N-doping. Chem. Commun. **55**, 14801-14804 (2019). https://doi.org/10.1039/c9cc08439h

[S12] S. Song, W. Li, Y.-P. Deng, Y. Ruan, Y. Zhang et al., TiC supported amorphous MnO_x as highly efficient bifunctional electrocatalyst for corrosion resistant oxygen electrode of Zn-air batteries. Nano Energy **67**, 104208 (2020). https://doi.org/10.1016/j.nanoen.2019.104208

[S13] M. Wu, G. Zhang, J. Qiao, N. Chen, W. Chen et al., Ultra-long life rechargeable Zinc-air battery based on high-performance trimetallic nitride and NCNT hybrid

bifunctional electrocatalysts. Nano Energy **61**, 86-95 (2019). https://doi.org/10.1016/j.nanoen.2019.04.031

[S14] Y. Fan, S. Ida, A. Staykov, T. Akbay, H. Hagiwara et al., Ni-Fe nitride nanoplates on nitrogen-doped graphene as a synergistic catalyst for reversible oxygen evolution reaction and rechargeable Zn-air battery. Small **13**, (2017). https://doi.org/10.1002/smll.201700099

[S15] Y.-N. Chen, Y. Guo, H. Cui, Z. Xie, X. Zhang et al., Bifunctional electrocatalysts of MOF-derived Co–N/C on bamboo-like MnO nanowires for high-performance liquid- and solid-state Zn–air batteries. J. Mater. Chem. A **6**, 9716-9722 (2018). https://doi.org/10.1039/c8ta01859f

[S16] X. Liu, L. Wang, P. Yu, C. Tian, F. Sun et al., A stable bifunctional catalyst for rechargeable zinc-air batteries: iron-cobalt nanoparticles embedded in a nitrogen-doped 3D carbon matrix. Angew. Chem. Int. Ed. **57**, 16166-16170 (2018). https://doi.org/10.1002/anie.201809009

[S17] Z. Zhang, Y. P. Deng, Z. Xing, D. Luo, S. Sy et al., "Ship in a Bottle" Design of highly efficient bifunctional electrocatalysts for long-lasting rechargeable Zn-air batteries. ACS Nano **13**, 7062-7072 (2019). https://doi.org/10.1021/acsnano.9b02315

[S18] H. Zhang, J. Zhang, Y. Li, H. Jiang, H. Jiang et al., Continuous oxygen vacancy engineering of the Co_3O_4 layer for an enhanced alkaline electrocatalytic hydrogen evolution reaction. J. Mater. Chem. A **7**, 13506-13510 (2019). https://doi.org/10.1039/c9ta03652k

[S19] H. Luo, W. J. Jiang, S. Niu, X. Zhang, Y. Zhang et al., Self-catalyzed growth of Co-N-C nanobrushes for efficient rechargeable Zn-air batteries. Small **16**, 2001171 (2020). https://doi.org/10.1002/smll.202001171

[S20] D. Ren, J. Ying, M. Xiao, Y. P. Deng, J. Ou et al., Hierarchically porous multimetal-based carbon nanorod hybrid as an efficient oxygen catalyst for rechargeable Zinc–air batteries. Adv. Funct. Mater. **30**, 1908167 (2019). https://doi.org/10.1002/adfm.201908167

[S21] H.-P. Guo, X.-W. Gao, N.-F. Yu, Z. Zheng, W.-B. Luo et al., Metallic state two-dimensional holey-structured Co_3FeN nanosheets as stable and bifunctional electrocatalysts for Zinc–air batteries. J. Mater. Chem. A **7**, 26549-26556 (2019). https://doi.org/10.1039/c9ta10079b

[S22] H. Lei, Z. Wang, F. Yang, X. Huang, J. Liu et al., NiFe nanoparticles embedded N-doped carbon nanotubes as high-efficient electrocatalysts for wearable solid-state Zn-air batteries. Nano Energy **68**, 104293 (2020). https://doi.org/10.1016/j.nanoen.2019.104293

[S23] G. Liu, J. Li, J. Fu, G. Jiang, G. Lui et al., An Oxygen-vacancy-rich semiconductor-supported bifunctional catalyst for efficient and stable Zinc-air

batteries. Adv. Mater. **31**, 1806761 (2019). https://doi.org/10.1002/adma.201806761

[S24] P. Zhu, J. Gao, S. Liu, Facile in situ coupling CoFe/Co nanoparticles and N-doped carbon nanotubes/graphitic nanosheets as bifunctional oxygen electrocatalysts for rechargeable Zn-air batteries. J. Power Sources **449**, 227512 (2020). https://doi.org/10.1016/j.jpowsour.2019.227512

[S25] P. Liu, D. Gao, W. Xiao, L. Ma, K. Sun et al., Self-powered water-splitting devices by core-shell NiFe@N-graphite-based Zn-air Batteries. Adv. Funct. Mater. **28**, 1706928 (2018). https://doi.org/10.1002/adfm.201706928

[S26]D. U. Lee, M. G. Park, H. W. Park, M. H. Seo, X. Wang et al., Highly active and durable nanocrystal-decorated bifunctional electrocatalyst for rechargeable Zinc-air batteries. ChemSusChem **8**, 3129-38 (2015). https://doi.org/10.1002/cssc.201500609

[S27] P. Yu, L. Wang, F. Sun, Y. Xie, X. Liu et al., Co Nanoislands rooted on Co-N-C Nanosheets as efficient oxygen electrocatalyst for Zn-air batteries. Adv. Mater. **31**, 1901666 (2019). https://doi.org/10.1002/adma.201901666
